# ADJUSTING TECHNOLOGY FOR AN AGEING POPULATION: CO-PRODUCING TECHNOLOGICAL SOLUTIONS WITH OLDER ADULTS.

**DOI:** 10.1093/geroni/igad104.3753

**Published:** 2023-12-21

**Authors:** Deborah Morgan, Hannah R Marston, Carol Maddock, Aelwyn Williams, Gemma Wilson- Menzfeld, Elizabeth Jones, Jessica Gates

**Affiliations:** Swansea University, Swansea, Wales, United Kingdom; Open University, Milton Keynes, England, United Kingdom; Swansea University, Swansea, Wales, United Kingdom; Swansea University, Swansea, Wales, United Kingdom; Northumbria University, Newcastle, England, United Kingdom; Swansea University, Swansea, Wales, United Kingdom; Northumbria University, Newcastle, England, United Kingdom

## Abstract

Since the onset of Covid 19, there has been a shift towards moving essential services online. This has the potential to further exclude sub-sections of the population from accessing essential services. This is particularly the case for older people who lack basic digital skills or who are not confident using digital technology, amplifying the risk of exclusion, and increasing the risk of social exclusion, loneliness and social isolation among digitally excluded older people. Research suggests that barriers to engagement with digital technologies include cost, lack of support, and technology being perceived as complicated or not for ‘people like me (French, Quinn & Yates, 2019). As such, there is an urgent need to develop digital solutions with older people, co-designing technology that meets their needs. The presentation will draw on data and experiences of older people involved in a seed corn project funded by the Research Wales Innovation Fund (April 2023- July 2023). The project aimed to co-design an affordable digital solution with digitally excluded older people to support the development of digital skills and confidence. The presentation will focus on the skills deficit identified by older adults with limited digital skills and explore how this knowledge is being incorporated into a co-designed prototype launcher app. The presentation will highlight the benefits and challenges of co-producing solutions for an ageing population.

